# 2-Amino-5-chloro­pyridinium 4-amino­benzoate

**DOI:** 10.1107/S1600536812043085

**Published:** 2012-10-20

**Authors:** V. Kannan, P. Sugumar, S. Brahadeeswaran, M. N. Ponnuswamy

**Affiliations:** a Department of Physics, M.A.M. School of Engineering, Siruganur, Tiruchirappalli 621 105, India; b Centre of Advanced Study in Crystallography and Biophysics, University of Madras, Guindy Campus, Chennai 600 025, India; c Department of Physics, Anna University, BIT Campus, Tiruchirappalli 620 024, India

## Abstract

In the title molecular salt, C_5_H_6_ClN_2_^+^·C_7_H_6_NO_2_^−^, the cations and anions are connected by cation-to-anion and anion-to-anion N—H⋯O hydrogen bonds into a three-dimensional network. The dihedral angle between the ring and the CO_2_ group in the anion is 7.14 (7)°.

## Related literature

For general background to chloro­pyridinium derivatives, see: Brahadeeswaran *et al.* (2006 ▶); Tomaru *et al.* (1991 ▶). For N—H⋯O hydrogen bonds, see: Blessing (1986 ▶); Brown (1976 ▶). 
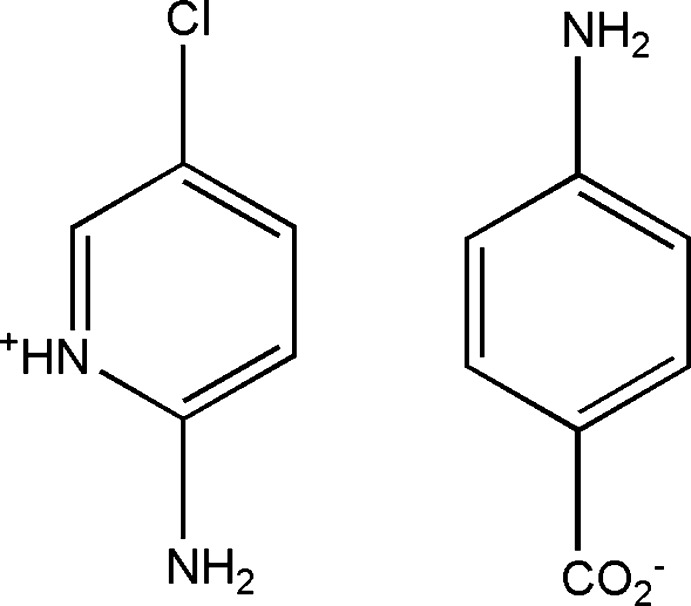


## Experimental

### 

#### Crystal data

C_5_H_6_ClN_2_^+^·C_7_H_6_NO_2_^−^
*M* *_r_* = 265.70
Monoclinic, 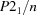

*a* = 6.9879 (4) Å
*b* = 22.0074 (13) Å
*c* = 8.0554 (5) Å
β = 92.796 (1)°
*V* = 1237.33 (13) Å^3^
*Z* = 4
Mo *K*α radiation
μ = 0.31 mm^−1^
*T* = 293 K
0.20 × 0.19 × 0.18 mm

#### Data collection

Bruker APEXII area-detector diffractometer
Absorption correction: multi-scan (*SADABS*; Bruker, 2008 ▶) *T*_min_ = 0.941, *T*_max_ = 0.946
12108 measured reflections
3086 independent reflections
2642 reflections with *I* > 2σ(*I*)
*R*_int_ = 0.021

#### Refinement

*R*[*F* ^2^ > 2σ(*F* ^2^)] = 0.038
*wR*(*F* ^2^) = 0.112
*S* = 1.04
3086 reflections
179 parameters
H atoms treated by a mixture of independent and constrained refinement
Δρ_max_ = 0.20 e Å^−3^
Δρ_min_ = −0.31 e Å^−3^

### 

Data collection: *APEX2* (Bruker, 2008 ▶); cell refinement: *SAINT* (Bruker, 2008 ▶); data reduction: *SAINT*; program(s) used to solve structure: *SHELXS97* (Sheldrick, 2008 ▶); program(s) used to refine structure: *SHELXL97* (Sheldrick, 2008 ▶); molecular graphics: *ORTEP-3* (Farrugia, 1997 ▶); software used to prepare material for publication: *SHELXL97* and *PLATON* (Spek, 2009 ▶).

## Supplementary Material

Crystal structure: contains datablock(s) global, I. DOI: 10.1107/S1600536812043085/bt6844sup1.cif

Structure factors: contains datablock(s) I. DOI: 10.1107/S1600536812043085/bt6844Isup2.hkl

Supplementary material file. DOI: 10.1107/S1600536812043085/bt6844Isup3.cml

Additional supplementary materials:  crystallographic information; 3D view; checkCIF report

## Figures and Tables

**Table 1:** Hydrogen-bond geometry (Å, °)

*D*—H⋯*A*	*D*—H	H⋯*A*	*D*⋯*A*	*D*—H⋯*A*
N1—H1⋯O2^i^	0.86	1.76	2.6135 (15)	175
N2—H2*A*⋯O1^i^	0.89 (2)	1.94 (2)	2.8216 (18)	172.1 (19)
N2—H2*B*⋯O1^ii^	0.86 (2)	2.10 (2)	2.8776 (17)	150.3 (19)
N3—H3*A*⋯O1^iii^	0.862 (19)	2.19 (2)	3.0357 (18)	167.4 (17)
N3—H3*B*⋯O2^iv^	0.86 (2)	2.08 (2)	2.9291 (18)	171 (2)

Symmetry codes: (i) 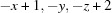; (ii) 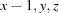; (iii) 
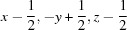
; (iv) 
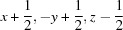
.

**Fig. 1.**
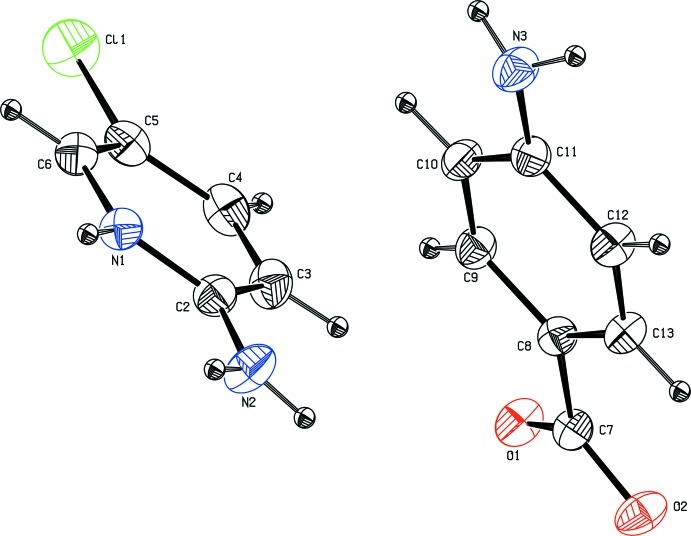
The molecular structure of the title compound, showing the atomic numbering and displacement ellipsoids drawn at 30% probability level.

**Fig. 2.**
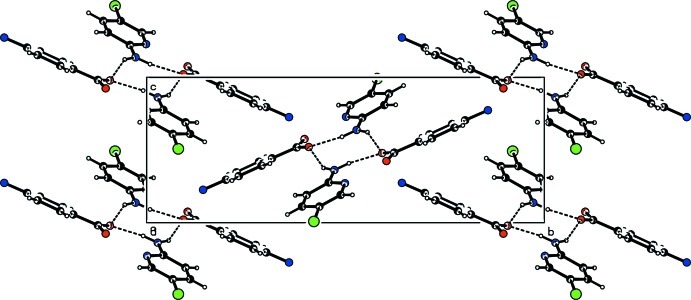
The crystal packing of the molecules viewed down a axis.

## supplementary crystallographic information

### Comment

Pyridine heterocycles and their derivatives are present in many large molecules having photo chemical, electro chemical and catalytic applications. Pyridine derivatives possess nonlinear optical (NLO) properties(Tomaru *et al.*, 1991). 4-*N*,*N*-dimethylamino-4'-*N*'-methyl stilbazolium tosylate (DAST) is used in generating and detecting terahertz (THz) frequencies (Brahadeeswaran *et al.*, 2006). An attempt is made to solve the pyridine based crystal structures to explore the NLO behaviour.

The *ORTEP* plot of the molecule is shown in Fig.1. The structure can be described as segregated (C_5_H_6_ClN_2_)^+^.(C_7_H_6_NO_2_)^-^ groups and connected *via* N—H···O hydrogen bonds (Blessing, 1986; Brown, 1976). The dihedral angle between the chloropyridinium ring and aminobenzoate group is 51.5 (7)°. The external bond angle [N1—C2—N2=] 118.1 (1)° at the attached amino group in pyridinium moiety is slightly widened due to the hydrogen bond formation between the ionic groups.

A dimer formation occurs through N—H···O hydrogen bonds between the symmetry related molecules(Fig.2). N—H···O type of hydrogen bonds stabilize the molecules in the unit cell.

### Experimental

Methanol solutions of 2-amino-5-chloropyridine (64.28 mg, Aldrich) and 4-aminobenzoic acid (68.57 mg, Merck) were mixed together and stirred for about 1 h to get a homogeneous mixture. The resulting solution was allowed to evaporate at 303 K slowly in a water bath which has a temperature accuracy of ± 0.01°C at ambient atmosphere. Brown colour crystals with developed morphology of title compound were obtained after 12 days.

### Refinement

H atoms bonded to aromatic C and N atoms were positioned geometrically (N—H = 0.86 Å and C—H = 0.93 Å) and allowed to ride on their parent atoms,with *U*_iso_(H) = 1.2*U*_eq_(C,N). The H atoms of the two NH_2_ groups were freely refined.

### Crystal data

**Table:**

C_5_H_6_ClN_2_^+^·C_7_H_6_NO_2_^−^	*F*(000) = 552
*M**_r_* = 265.70	*D*_x_ = 1.426 Mg m^−^^3^
Monoclinic, *P*2_1_/*n*	Mo *K*α radiation, λ = 0.71073 Å
Hall symbol: -P 2yn	Cell parameters from 2642 reflections
*a* = 6.9879 (4) Å	θ = 1.9–28.4°
*b* = 22.0074 (13) Å	µ = 0.31 mm^−^^1^
*c* = 8.0554 (5) Å	*T* = 293 K
β = 92.796 (1)°	Block, white crystalline
*V* = 1237.33 (13) Å^3^	0.20 × 0.19 × 0.18 mm
*Z* = 4	

### Data collection

**Table:**

Bruker APEXII area-detector diffractometer	3086 independent reflections
Radiation source: fine-focus sealed tube	2642 reflections with *I* > 2σ(*I*)
Graphite monochromator	*R*_int_ = 0.021
ω scan	θ_max_ = 28.4°, θ_min_ = 1.9°
Absorption correction: multi-scan (*SADABS*; Bruker, 2008)	*h* = −9→9
*T*_min_ = 0.941, *T*_max_ = 0.946	*k* = −29→29
12108 measured reflections	*l* = −10→10

### Refinement

**Table:**

Refinement on *F*^2^	Primary atom site location: structure-invariant direct methods
Least-squares matrix: full	Secondary atom site location: difference Fourier map
*R*[*F*^2^ > 2σ(*F*^2^)] = 0.038	Hydrogen site location: inferred from neighbouring sites
*wR*(*F*^2^) = 0.112	H atoms treated by a mixture of independent and constrained refinement
*S* = 1.04	*w* = 1/[σ^2^(*F*_o_^2^) + (0.0572*P*)^2^ + 0.3359*P*] where *P* = (*F*_o_^2^ + 2*F*_c_^2^)/3
3086 reflections	(Δ/σ)_max_ = 0.001
179 parameters	Δρ_max_ = 0.20 e Å^−^^3^
0 restraints	Δρ_min_ = −0.31 e Å^−^^3^

### Special details

**Table:**

Geometry. All e.s.d.'s (except the e.s.d. in the dihedral angle between two l.s. planes) are estimated using the full covariance matrix. The cell e.s.d.'s are taken into account individually in the estimation of e.s.d.'s in distances, angles and torsion angles; correlations between e.s.d.'s in cell parameters are only used when they are defined by crystal symmetry. An approximate (isotropic) treatment of cell e.s.d.'s is used for estimating e.s.d.'s involving l.s. planes.
Refinement. Refinement of *F*^2^ against ALL reflections. The weighted *R*-factor *wR* and goodness of fit *S* are based on *F*^2^, conventional *R*-factors *R* are based on *F*, with *F* set to zero for negative *F*^2^. The threshold expression of *F*^2^ > σ(*F*^2^) is used only for calculating *R*-factors(gt) *etc*. and is not relevant to the choice of reflections for refinement. *R*-factors based on *F*^2^ are statistically about twice as large as those based on *F*, and *R*- factors based on ALL data will be even larger.

### Fractional atomic coordinates and isotropic or equivalent isotropic displacement parameters (Å^2^)

**Table:**

	*x*	*y*	*z*	*U*_iso_*/*U*_eq_	
C2	0.3159 (2)	0.04218 (6)	0.77668 (17)	0.0407 (3)	
C3	0.3355 (2)	0.09763 (7)	0.6908 (2)	0.0479 (3)	
H3	0.2372	0.1261	0.6889	0.057*	
C4	0.4987 (2)	0.10919 (7)	0.61093 (19)	0.0486 (4)	
H4	0.5133	0.1460	0.5563	0.058*	
C5	0.6448 (2)	0.06560 (7)	0.61107 (17)	0.0431 (3)	
C6	0.6216 (2)	0.01208 (6)	0.69117 (17)	0.0406 (3)	
H6	0.7173	−0.0173	0.6911	0.049*	
C7	0.71327 (19)	0.12257 (6)	1.01810 (16)	0.0359 (3)	
C8	0.71041 (18)	0.18462 (6)	0.94334 (16)	0.0346 (3)	
C9	0.87870 (19)	0.21125 (6)	0.89231 (17)	0.0389 (3)	
H9	0.9937	0.1902	0.9066	0.047*	
C10	0.87784 (19)	0.26832 (6)	0.82089 (17)	0.0407 (3)	
H10	0.9923	0.2854	0.7895	0.049*	
C11	0.70654 (19)	0.30071 (6)	0.79533 (16)	0.0371 (3)	
C12	0.5373 (2)	0.27406 (6)	0.84646 (18)	0.0412 (3)	
H12	0.4219	0.2948	0.8311	0.049*	
C13	0.54024 (19)	0.21735 (6)	0.91937 (17)	0.0390 (3)	
H13	0.4264	0.2006	0.9533	0.047*	
N1	0.45860 (17)	0.00142 (5)	0.77144 (14)	0.0385 (3)	
H1	0.4461	−0.0329	0.8210	0.046*	
N2	0.1643 (2)	0.02799 (7)	0.86228 (19)	0.0545 (4)	
N3	0.7058 (2)	0.35808 (6)	0.72634 (18)	0.0477 (3)	
O1	0.86491 (14)	0.09238 (5)	1.02101 (14)	0.0471 (3)	
O2	0.55877 (14)	0.10303 (4)	1.07566 (14)	0.0483 (3)	
Cl1	0.85224 (7)	0.07952 (2)	0.50832 (6)	0.06516 (16)	
H3A	0.598 (3)	0.3696 (9)	0.680 (2)	0.049 (5)*	
H2A	0.155 (3)	−0.0086 (11)	0.909 (2)	0.067 (6)*	
H2B	0.078 (3)	0.0551 (10)	0.877 (3)	0.069 (6)*	
H3B	0.808 (3)	0.3657 (9)	0.676 (3)	0.062 (5)*	

### Atomic displacement parameters (Å^2^)

**Table:**

	*U*^11^	*U*^22^	*U*^33^	*U*^12^	*U*^13^	*U*^23^
C2	0.0455 (7)	0.0342 (6)	0.0423 (7)	0.0007 (5)	0.0014 (5)	−0.0021 (5)
C3	0.0525 (8)	0.0348 (7)	0.0557 (8)	0.0043 (6)	−0.0035 (7)	0.0047 (6)
C4	0.0605 (9)	0.0358 (7)	0.0488 (8)	−0.0081 (6)	−0.0060 (7)	0.0091 (6)
C5	0.0458 (7)	0.0437 (7)	0.0395 (7)	−0.0108 (6)	−0.0008 (5)	0.0013 (6)
C6	0.0414 (7)	0.0371 (7)	0.0432 (7)	−0.0019 (5)	0.0007 (5)	−0.0009 (5)
C7	0.0370 (6)	0.0310 (6)	0.0397 (6)	0.0004 (5)	0.0026 (5)	−0.0017 (5)
C8	0.0366 (6)	0.0310 (6)	0.0364 (6)	−0.0006 (5)	0.0032 (5)	−0.0013 (5)
C9	0.0330 (6)	0.0389 (7)	0.0447 (7)	0.0017 (5)	0.0026 (5)	0.0013 (5)
C10	0.0349 (6)	0.0418 (7)	0.0457 (7)	−0.0061 (5)	0.0048 (5)	0.0027 (6)
C11	0.0417 (7)	0.0319 (6)	0.0377 (6)	−0.0027 (5)	0.0014 (5)	−0.0015 (5)
C12	0.0366 (7)	0.0360 (6)	0.0512 (8)	0.0044 (5)	0.0050 (6)	0.0026 (6)
C13	0.0346 (6)	0.0359 (6)	0.0470 (7)	−0.0019 (5)	0.0071 (5)	0.0008 (5)
N1	0.0444 (6)	0.0297 (5)	0.0414 (6)	−0.0003 (4)	0.0041 (5)	0.0025 (4)
N2	0.0529 (8)	0.0424 (7)	0.0698 (9)	0.0093 (6)	0.0199 (7)	0.0069 (6)
N3	0.0450 (7)	0.0381 (6)	0.0600 (8)	−0.0030 (5)	0.0026 (6)	0.0104 (6)
O1	0.0389 (5)	0.0374 (5)	0.0654 (7)	0.0055 (4)	0.0072 (5)	0.0045 (4)
O2	0.0403 (5)	0.0372 (5)	0.0686 (7)	0.0042 (4)	0.0144 (5)	0.0137 (5)
Cl1	0.0575 (3)	0.0704 (3)	0.0685 (3)	−0.0175 (2)	0.0128 (2)	0.0112 (2)

### Geometric parameters (Å, º)

**Table:**

C2—N2	1.329 (2)	C8—C13	1.3956 (18)
C2—N1	1.3433 (18)	C9—C10	1.3814 (19)
C2—C3	1.413 (2)	C9—H9	0.9300
C3—C4	1.360 (2)	C10—C11	1.3997 (19)
C3—H3	0.9300	C10—H10	0.9300
C4—C5	1.401 (2)	C11—N3	1.3794 (18)
C4—H4	0.9300	C11—C12	1.3997 (19)
C5—C6	1.357 (2)	C12—C13	1.3791 (19)
C5—Cl1	1.7312 (15)	C12—H12	0.9300
C6—N1	1.3573 (17)	C13—H13	0.9300
C6—H6	0.9300	N1—H1	0.8600
C7—O1	1.2500 (16)	N2—H2A	0.89 (2)
C7—O2	1.2706 (16)	N2—H2B	0.86 (2)
C7—C8	1.4921 (18)	N3—H3A	0.862 (19)
C8—C9	1.3937 (18)	N3—H3B	0.86 (2)
			
N2—C2—N1	118.11 (13)	C8—C9—H9	119.4
N2—C2—C3	123.76 (14)	C9—C10—C11	120.70 (12)
N1—C2—C3	118.14 (13)	C9—C10—H10	119.6
C4—C3—C2	119.78 (14)	C11—C10—H10	119.6
C4—C3—H3	120.1	N3—C11—C10	120.77 (13)
C2—C3—H3	120.1	N3—C11—C12	121.04 (13)
C3—C4—C5	119.95 (13)	C10—C11—C12	118.16 (12)
C3—C4—H4	120.0	C13—C12—C11	120.62 (12)
C5—C4—H4	120.0	C13—C12—H12	119.7
C6—C5—C4	119.40 (14)	C11—C12—H12	119.7
C6—C5—Cl1	120.23 (12)	C12—C13—C8	121.38 (12)
C4—C5—Cl1	120.37 (11)	C12—C13—H13	119.3
C5—C6—N1	119.95 (13)	C8—C13—H13	119.3
C5—C6—H6	120.0	C2—N1—C6	122.74 (12)
N1—C6—H6	120.0	C2—N1—H1	118.6
O1—C7—O2	123.22 (12)	C6—N1—H1	118.6
O1—C7—C8	119.25 (12)	C2—N2—H2A	120.4 (13)
O2—C7—C8	117.53 (11)	C2—N2—H2B	119.4 (14)
C9—C8—C13	117.89 (12)	H2A—N2—H2B	120.1 (19)
C9—C8—C7	120.59 (12)	C11—N3—H3A	115.5 (12)
C13—C8—C7	121.51 (12)	C11—N3—H3B	112.7 (14)
C10—C9—C8	121.23 (12)	H3A—N3—H3B	117.8 (18)
C10—C9—H9	119.4		
			
N2—C2—C3—C4	177.79 (15)	C7—C8—C9—C10	179.13 (12)
N1—C2—C3—C4	−2.3 (2)	C8—C9—C10—C11	−1.0 (2)
C2—C3—C4—C5	1.4 (2)	C9—C10—C11—N3	179.01 (13)
C3—C4—C5—C6	0.1 (2)	C9—C10—C11—C12	1.0 (2)
C3—C4—C5—Cl1	179.43 (12)	N3—C11—C12—C13	−178.26 (13)
C4—C5—C6—N1	−0.6 (2)	C10—C11—C12—C13	−0.2 (2)
Cl1—C5—C6—N1	−179.96 (10)	C11—C12—C13—C8	−0.5 (2)
O1—C7—C8—C9	−6.38 (19)	C9—C8—C13—C12	0.5 (2)
O2—C7—C8—C9	173.83 (12)	C7—C8—C13—C12	−178.37 (13)
O1—C7—C8—C13	172.44 (13)	N2—C2—N1—C6	−178.27 (13)
O2—C7—C8—C13	−7.35 (19)	C3—C2—N1—C6	1.8 (2)
C13—C8—C9—C10	0.3 (2)	C5—C6—N1—C2	−0.4 (2)

### Hydrogen-bond geometry (Å, º)

**Table:**

*D*—H···*A*	*D*—H	H···*A*	*D*···*A*	*D*—H···*A*
N1—H1···O2^i^	0.86	1.76	2.6135 (15)	175
N2—H2*A*···O1^i^	0.89 (2)	1.94 (2)	2.8216 (18)	172.1 (19)
N2—H2*B*···O1^ii^	0.86 (2)	2.10 (2)	2.8776 (17)	150.3 (19)
N3—H3*A*···O1^iii^	0.862 (19)	2.19 (2)	3.0357 (18)	167.4 (17)
N3—H3*B*···O2^iv^	0.86 (2)	2.08 (2)	2.9291 (18)	171 (2)

Symmetry codes: (i) −*x*+1, −*y*, −*z*+2; (ii) *x*−1, *y*, *z*; (iii) *x*−1/2, −*y*+1/2, *z*−1/2; (iv) *x*+1/2, −*y*+1/2, *z*−1/2.
